# Nutritional status in female patients with nontuberculous mycobacterial lung disease and its association with disease severity

**DOI:** 10.1186/s12890-022-02109-5

**Published:** 2022-08-15

**Authors:** Yumi Takayama, Takamasa Kitajima, Noritsugu Honda, Naoki Sakane, Yukina Yumen, Motonari Fukui, Narumi Nagai

**Affiliations:** 1 Department of Nutrition, Tazuke Kofukai Medical Research Institute, Kitano Hospital, 2-4-20 Ogi-machi, Kita-ku, Osaka-city, Osaka 530-8480 Japan; 2 Respiratory Disease Center, Tazuke Kofukai Medical Research Institute, Kitano Hospital, 2-4-20 Ogi-machi, Kita-ku, Osaka-city, Osaka 530-8480 Japan; 3 Department of Rehabilitation, Tazuke Kofukai Medical Research Institute, Kitano Hospital, 2-4-20 Ogi-machi, Kita-ku, Osaka-city, Osaka 530-8480 Japan; 4 Clinical Research Institute for Endocrine and Metabolic Disease, National Hospital Organization, Kyoto Medical Center, 1-1 Fukakusamukaihata-cho, Fushimi-ku, Kyoto-city, Kyoto 612-8555 Japan; 5 Graduate School of Human Science and Environment, University of Hyogo, 1-1-12 Shinzaike-honcho, Himeji city, Hyogo 670-0092 Japan

**Keywords:** Nontuberculous mycobacteria, Women, Dietary survey, Energy intake, Body habitus, Skeletal muscle, Prealbumin

## Abstract

**Background:**

In women, slender body habitus has been reported to be one of the predisposing factors underlying the development and poor prognosis of non-tuberculous mycobacterial lung disease (NTM-LD). Given the lack of nutritional data contributing to treatment strategies, we aimed to clarify the nutritional status of female patients with NTM-LD and its association with disease severity.

**Methods:**

In this single-center observational study, we enrolled 81 female outpatients with NTM-LD. Data on healthy women of similar ages were selected from our previous survey data and categorized as controls. First, we compared anthropometric and dietary survey data between patients and controls. Second, after the patients were categorized into relatively mild (mild, n = 40) and relatively severe groups (severe, n = 41) based on pulmonary X-ray-image finding scores, body composition, nutritional intake, and biochemical markers were compared between the groups. To identify nutritional factors associated with disease severity, logistic regression analyses were performed.

**Results:**

Compared with controls, patients with NTM-LD had significantly lower energy intake, body mass index, body fat, and skeletal muscle mass (all *p* < 0.001). Compared with the mild group, the severe group had significantly lower skeletal muscle mass (*p* = 0.037), albumin (*p* = 0.029), transthyretin (prealbumin) (*p* = 0.002), retinol-binding protein (*p* = 0.011), and hemoglobin (*p* = 0.001); however, no between-group differences were observed in energy or nutrient intake. Logistic analyses revealed that transthyretin (*p* = 0.025) and hemoglobin (*p* = 0.003) levels were independent factors associated with disease severity.

**Conclusions:**

This is the first study to comprehensively report the association between NTM-LD severity and nutritional status, including body composition, nutrient intake, and biomarkers. The results suggest that initiating nutritional therapy from the mild stage of the disease to prevent undernutrition is warranted.

**Supplementary Information:**

The online version contains supplementary material available at 10.1186/s12890-022-02109-5.

## Introduction

Nontuberculous mycobacterial lung disease (NTM-LD) is an infection caused by a group of bacteria that are naturally found in dust, soil, and water, and the incidence of NTM-LD has been increasing in various regions of the world [1].

It is known that NTM-LD occurs more commonly in thin women over 50 years of age [2–6] and that, compared to the general female population, female patients with NTM-LD have a slender body habitus [5, 6]. However, the reason underlying weight loss in female patients with NTM-LD remains unclear. Therefore, it is necessary to compare the nutritional intake of female patients with NTM-LD with that of the general female population using the same dietary survey methods; nonetheless, to the best of our knowledge, such data have not yet been reported. Moreover, regarding anthropometric characteristics, female patients with NTM-LD have been reported to have lower body weight and body fat than the general female population [5, 6]; however, the amount of skeletal muscle mass that correlates with respiratory muscle strength has not been fully elucidated. Therefore, whether female patients with NTM-LD have lower energy and nutrient intakes and are thinner with less muscle mass than the general female population warrants investigation.

Regarding the relationship between nutritional status and NTM-LD severity, several studies have demonstrated that poor nutritional status characterized by low body mass index (BMI) [7, 8], a thinner layer of chest subcutaneous fat [7], and low abdominal fat ratio [9] was associated with the progression of NTM-LD. Moreover, anemia [10], diminished lymphocyte count (< 1000 cells/μL) [11], and albumin levels (< 3.5 g/dL) [10–12] have been recognized as prognostic factors for mortality in patients with NTM-LD. The above findings imply that undernutrition aggravates the severity of NTM-LD. In this regard, nutritional therapy may be beneficial for treating and preventing disease severity. Nevertheless, there is a paucity of studies examining the relationship of nutritional status, such as dietary intake, long-term energy balance (degree of weight loss from 20 years of age), appetite, skeletal muscle mass, and NTM-LD severity.

Based on the above, the objectives of this study were to (1) determine the nutritional characteristics of female patients with NTM-LD in comparison with those of similar-aged, healthy women, and (2) explore the nutritional factors associated with NTM-LD severity. The goal of this study was to obtain data that can contribute to the implementation of nutritional therapy in patients with NTM-LD.

## Methods

### Participants

**Fig. 1:**
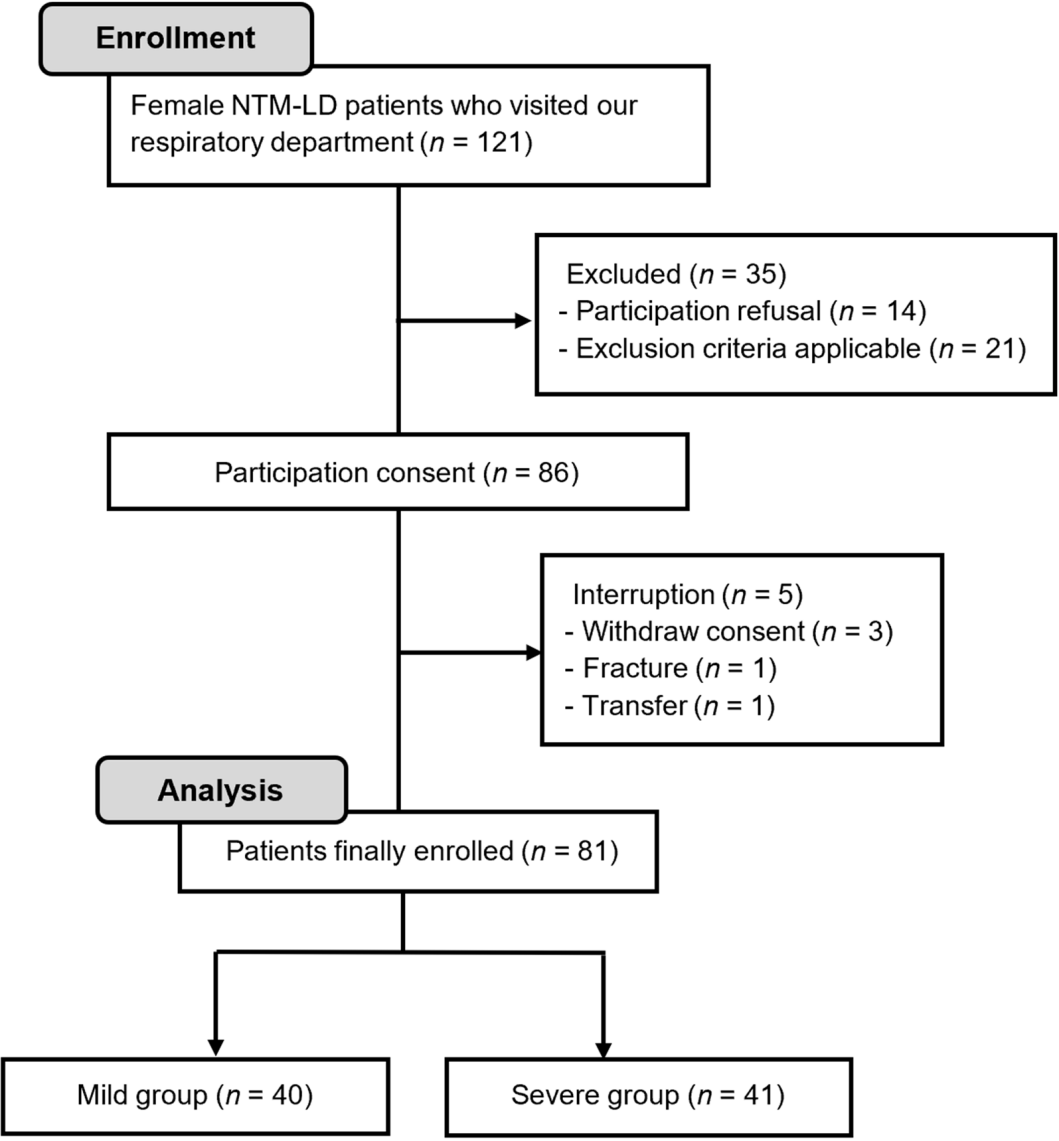
Patient enrollment diagram. *NTM-LD,* nontuberculous mycobacterial lung disease. Patients were classified into mild (score < 8.0) and severe (score ≥ 8.0) groups based on medical condition scores calculated using pulmonary X-ray image findings

This single-center observational study included 121 female outpatients with NTM-LD who were over 20 years of age and met the diagnostic criteria for NTM-LD, as stated by the American Thoracic Society (ATS) and Infectious Diseases Society of America (IDSA) [13] from the Respiratory Disease Center of Kitano Hospital (Osaka, Japan), between July and October 2018. Patients previously diagnosed with dementia or psychiatric disorders by a physician were excluded. Prior to inclusion, all participants received a detailed explanation of the study and subsequently provided written informed consent. As shown in Fig. 1, total of 81 patients were included in the analysis. To classify the severity of NTM-LD, we employed the NICE scoring system (ranging from 0 to 96) [14] which scores nodule (N), infiltration or consolidation (I), cavity (C), and ectasis (E) using chest radiographic images via a simple and versatile method. After the radiologist confirmed the presence of pulmonary and bronchial lesions, one respiratory physician read and scored the images. Two respiratory physicians then performed the confirmation process. All the respiratory physicians were certified by the Japanese Respiratory Society and had over 10 years of clinical experience. Based on the NICE score, patients were divided into relatively mild (mild, < 8.0, *n* = 40) or relatively severe (severe, ≥ 8.0, *n* = 41) group using a median value of 8.0.

To compare anthropometric and nutritional data between female patients with NTM-LD and the general female population, we used our previous survey data as control [15–17]. These data were obtained from individuals who attended a weekly stretching class at a public liberal art school in Himeji city. We selected control participants (*n* = 75) who possessed similar demographic background as the patients with NTM-LD (Japanese, women, mostly elderly, mostly living in urban area). Those who received care authorization or were diagnosed with dementia, cancer, or heart disease by a physician were excluded. All measurements were performed in Himeji Human science and Environment Campus of the University of Hyogo from April 2015 to September 2017 using entirely the same methodology of anthropometric and dietary survey.

This study was approved by the research ethics board of Kitano Hospital (Number: P180600300, May 1, 2018). The study procedures were performed in accordance with the Declaration of Helsinki and Clinical Practice Guidelines of the national government. Written informed consent was obtained from all participants before enrollment. This study was registered at the University Hospital Medical Information Network Center in Japan (trial registration UMIN000033689; August 8, 2018; www.umin.ac.jp).

### Measurements

#### Clinical measurements

Prior to obtaining measurements, patients’ medical records, including demographic and clinical data, were reviewed. On the day of the hospital visit, each patient underwent clinical measurements and nutritional assessments. Respiratory muscle strength (PEmax and Plmax) was measured using an electronic diagnostic spirometer (Autospiro AS-507 and AAM377, Minato Medical Science Co., Osaka, Japan). Albumin, lymphocyte count, total cholesterol, transthyretin (prealbumin), transferrin, retinol-binding protein, hemoglobin, and C-reactive protein levels were measured using blood sampled from the cubital veins.

#### Anthropometric and nutritional assessments

Height, weight, and body composition (InBody S10, BioSpace Co., Seoul, Korea) were measured on the same day as the clinical measurements. To assess the extent of weight loss, participants were asked about their maximum body weight and body weight at 20 years of age.

Dietary intake was estimated using a 24-h recall method for one typical weekday. The records were carefully assessed by registered dieticians via an interview with each participant. Nutritional values were calculated using computer-assisted procedures (Excell Eiyokun, ver. 8.2; Kenpakusya Co., Tokyo, Japan) based on the Japanese food consumption table [18]. Appetite scores were evaluated using the Japanese version of the Simplified Nutritional Appetite Questionnaire (SNAQ-J), consisting of four questions (appetite, satiety, taste, and frequency of meals), with a total score ranging from 4 to 20 and a higher score indicating better appetite [19]. Physical activity was evaluated using a shortened version of the International standardized Physical Activity Questionnaire (IPAQ) [20].

### Statistical analyses

All values are expressed as means ± standard deviations. All statistical analyses were performed using the Statistical Package for the Social Sciences (SPSS for Windows™ ver24; IBM Inc., Tokyo, Japan). Prior to statistical evaluation, normality testing was conducted using the Kolmogorov–Smirnov test.

We performed two comparisons: (1) between the patients and the general population, and (2) between two patient groups with different severity stages. Both comparisons were performed using a paired Student’s *t*-test or Welch’s *t*-test, as appropriate. For the comparison of patients and controls, two analyses were performed using data from all participants (Table 1). Sensitivity analysis was also performed: patients were matched 1:1 using R package “optmach,” distance-based bipartite matching by age (Additional file 1: Table S1). The associations between NICE scores and nutritional biomarkers were evaluated using Spearman correlation coefficients. To identify nutritional factors associated with disease severity, logistic regression analysis (adjusted for age, BMI, and energy intake) was performed. Albumin, transthyretin, retinol-binding protein, and hemoglobin were used as explanatory variables, and low (0) or high (1) NICE score was used as the response variable. The sample size could not be determined due to lack of a previous study. Patients with missing data were excluded from the relevant analysis. *p* values < 0.05 were considered statistically significant.

**Table 1:** Anthropometric and nutritional data of female patients with NTM-LD and healthy women

	NTM-LD	Control	*p* Value ^a^
	(*n* = 81)	(*n* = 75)	
Age (years)	70.8 ± 8.6	71.1 ± 2.9	0.794
*Anthropometric data*			
Height (cm)	153.7 ± 6.9	152.9 ± 5.1	0.436
Body weight (kg)	46.8 ± 7.2	53.3 ± 7.8	< 0.001
Body mass index (kg/m^2^)	19.8 ± 2.5	22.8 ± 2.9	< 0.001
Fat mass (kg)	12.8 ± 4.8	16.5 ± 5.4	< 0.001
Percentage of body fat (%)	26.7 ± 7.1	30.4 ± 6.1	0.001
Skeletal muscle mass (kg)	17.8 ± 2.5	19.6 ± 2.1	< 0.001
*Nutritional data*			
Energy (kcal/day)	1673 ± 340	1926 ± 375	< 0.001
Protein (g/day)	64.2 ± 18.0	79.4 ± 18.9	< 0.001
Fat (g/day)	51.2 ± 17.4	59.9 ± 17.3	0.002
Carbohydrate (g/day)	229.9 ± 55.7	260.0 ± 60.3	0.002

Mean ± standard deviation

*NTM-LD* nontuberculous mycobacterial lung disease

^a^Unpaired *t*-test

## Results

### Anthropometric and nutritional characteristics of female patients with NTM-LD

Table 1 presents the anthropometric and nutritional characteristics of female patients with NTM-LD and similar-aged, healthy women. Compared with controls, female patients with NTM-LD exhibited a distinct anthropometric profile, including significantly lower body weight, BMI, fat mass, and skeletal muscle mass (all *p* < 0.001). Similarly, in terms of nutritional profile, female patients with NTM-LD had significantly lower energy (*p* < 0.001), protein (*p* < 0.001), fat (*p* = 0.002), and carbohydrate (*p* = 0.002) intakes than healthy controls. The age-matched analysis of patients (*n* = 38) and controls (*n* = 38) showed the same results (Additional file 1: Table S1).

### Demographic characteristics of the mild and severe groups

Table 2 presents the demographic and clinical characteristics of the patient groups classified according to NICE scores. Compared with the mild group, the severe group tended to be older (*p* = 0.060) and have a longer history of illness (*p* = 0.053). Compared with the mild group, the severe group also had a higher frequency of fibrocavitary lesions (*p* = 0.040), cavity formation (*p* = 0.002), and number of patients using two or more antibiotic drugs (*p* = 0.009). As indicators of respiratory muscle strength, significantly lower PEmax and PImax were observed (*p* = 0.001).

**Table 2:** Demographic and clinical characteristics of female patients with NTM-LD

Variables	All	Disease severity		*p* Value^a^
		Mild	Severe	
	(*n* = 81)	(*n* = 40)	(*n* = 41)	
Age (years)	70.8 ± 8.6	69.0 ± 7.0	72.6 ± 9.6	0.060
Smoking history (pack·year)	0.2 ± 1.2	0.3 ± 1.1	0.2 ± 1.2	0.762
Duration of NTM-LD (month)	72.3 ± 56.0	51.8 ± 41.9	92.9 ± 61.1	0.053
*Comorbidity*				
Malignancy (includes lung cancer)	17 (21.0)	9 (22.5)	8 (19.5)	0.790
Respiratory diseases	5 (6.2)	2 (5.0)	3 (7.3)	1.000
Diabetes mellitus	4 (4.9)	0 (0)	4 (9.8)	0.116
Cardiac diseases	8 (9.9)	4 (10.0)	4 (9.8)	1.000
Kidney diseases	2 (2.5)	0 (0)	2 (4.9)	0.494
Liver diseases	4 (4.9)	3 (7.5)	1 (2.4)	0.359
*Radiological findings*^*b*^				
NB	60 (74.1)	36 (90.0)	24 (58.5)	0.040
CavitaryNB	18 (22.2)	4 (10.0)	14 (34.1)	
FC	3 (3.7)	0 (0)	3 (7.3)	
With cavity	21 (25.9)	4 (10.0)	17 (41.5)	0.002
*NTM species*				
*M. avium*	51 (63.0)	28 (70.0)	23 (56.1)	0.252
*M. intracellulare*	28 (34.6)	11 (27.5)	17 (41.5)	0.244
*M. kansasii*	1 (1.2)	1 (2.5)	0 (0)	0.494
*M. abscessus*	7 (8.6)	2 (5.0)	5 (12.2)	0.432
*Antibiotic treatments*^*b*^				
Observation	17 (21.0)	14 (35.0)	3 (7.3)	0.009
One drug	7 (8.6)	3 (7.5)	4 (9.8)	
Two or more drugs	57 (70.4)	23 (57.5)	34 (82.9)	
*Respiratory muscle strength*				
PEmax (cmH_2_O)^c^	53.5 ± 23.9	62.8 ± 24.1	44.2 ± 20.1	0.001
PImax (cmH_2_O)^d^	32.2 ± 13.5	37.1 ± 14.9	27.2 ± 9.8	0.001
*Blood biochemistry*				
CRP (mg/dL)	0.31 ± 0.73	0.18 ± 0.40	0.43 ± 0.94	0.119

Mean ± standard deviation or *n* (%)

*NTM-LD* nontuberculous mycobacterial lung disease, *NB* nodular bronchiectatic type, *FC* fibrocavitary type, *CRP* C-reactive protein

^a^Unpaired *t*-test (Mild vs. Severe)

^b^χ^2^ test or Fisher’s exact test

^c^All; n = 74, Mild; n = 37, Severe; n = 37

^d^All; n = 73, Mild; n = 37, Severe; n = 36

### Comparison of anthropometric and nutritional data between mild and severe groups

Table 3 presents the values of the anthropometric measurements, nutritional intakes, lifestyle indicators, and blood biochemistry of the patients. The severe group had significantly lower body weight (*p* = 0.016) and skeletal muscle mass (*p* = 0.037) than the mild group. BMI (*p* = 0.079) and fat mass (*p* = 0.050) tended to be lower in the severe group than in the mild group. There were no differences in longitudinal weight loss, appetite scores, and physical activity between the two groups. No significant between-group differences were observed in energy and nutrient intakes, indicating that the average energy intake was equivalent between the groups. Blood biochemistry assessments revealed that albumin (*p* = 0.029), transthyretin (*p* = 0.002), retinol-binding protein (*p* = 0.011), and hemoglobin (*p* = 0.001) levels were significantly lower in the severe group than in the mild group.

**Table 3:** Anthropometric and nutritional characteristics of female patients with NTM-LD

Variables	All	Disease severity		*p* Value^a^
		Mild	Severe	
	(*n* = 81)	(*n* = 40)	(*n* = 41)	
*Anthropometric data*				
Height (cm)	153.7 ± 6.9	155.0 ± 6.6	152.5 ± 7.1	0.101
Body weight (kg)	46.8 ± 7.2	48.7 ± 6.7	44.9 ± 7.3	0.016
Body mass index (kg/m^2^)	19.8 ± 2.5	20.3 ± 2.3	19.3 ± 2.6	0.079
Fat mass (kg)	12.8 ± 4.8	13.9 ± 4.5	11.8 ± 4.9	0.050
Percentage of body fat (%)	26.7 ± 7.1	27.9 ± 6.6	25.5 ± 7.5	0.137
Skeletal muscle mass (kg)	17.8 ± 2.5	18.3 ± 2.3	17.2 ± 2.6	0.037
Weight loss from 20 years of age (kg)	1.9 ± 7.2	1.5 ± 6.9	2.3 ± 7.6	0.638
Percentage weight loss from 20 years of age (%)	3.3 ± 14.8	2.4 ± 13.3	4.3 ± 16.2	0.579
Weight loss from maximum body weight (kg)	7.7 ± 6.1	7.2 ± 6.7	8.2 ± 5.5	0.481
Percentage weight loss from maximum body weight (%)	13.9 ± 10.1	12.4 ± 10.2	15.4 ± 9.9	0.180
*Nutritional intake*				
Energy (kcal/day)	1673 ± 340	1666 ± 362	1680 ± 321	0.850
Protein (g/day)	64.2 ± 18.0	63.6 ± 19.3	64.9 ± 16.9	0.748
Protein (g/day/1000 kcal)	38.2 ± 6.7	37.8 ± 6.7	38.6 ± 6.7	0.618
Fat (g/day)	51.2 ± 17.4	52.4 ± 18.5	50.0 ± 16.3	0.534
Fat (g/day/1000 kcal)	30.4 ± 7.7	31.3 ± 8.0	29.5 ± 7.3	0.290
Carbohydrate (g/day)	229.9 ± 55.7	226.2 ± 58.6	233.5 ± 53.3	0.558
Carbohydrate (g/day/1000 kcal)	138.2 ± 21.5	136.5 ± 22.2	139.9 ± 21.0	0.475
Appetite score (SNAQ-J)	14.6 ± 1.7	14.8 ± 1.6	14.4 ± 1.7	0.302
Amount of physical activity (MET·min/week)	1806 ± 2410	2074 ± 2754	1538 ± 2007	0.323
Duration of sitting time (min/day)	289 ± 162	284 ± 140	294 ± 183	0.770
*Blood biochemistry*				
Albumin (g/dL)	4.4 ± 0.3	4.4 ± 0.3	4.3 ± 0.3	0.029
Transthyretin (mg/dL)^b^	20.0 ± 4.5	21.5 ± 3.5	18.4 ± 4.9	0.002
Transferrin (mg/dL)	224 ± 37	230 ± 34	218 ± 40	0.157
Retinol-binding protein (mg/dL)^b^	2.5 ± 0.6	2.7 ± 0.5	2.3 ± 0.6	0.011
Total cholesterol (mg/dL)	225 ± 36	226 ± 33	225 ± 38	0.886
Hemoglobin (g/dL)	13.1 ± 1.1	13.5 ± 1.0	12.7 ± 1.0	0.001
Lymphocyte count (× 10^2^/μL)	13.4 ± 4.0	13.8 ± 3.4	13.1 ± 4.5	0.415

Mean ± standard deviation

*NTM-LD* nontuberculous mycobacterial lung disease

^a^Unpaired *t-*test (Mild vs. Severe)

^b^Data (transthyretin and retinol-binding protein) of one severe patient were not obtained

### Correlations between NICE scores and biochemical markers

Transthyretin (*r* =  − 0.348, *p* = 0.002), retinol-binding protein (*r* =  − 0.264, *p* = 0.018), and hemoglobin (*r* =  − 0.280, *p* = 0.011) levels were negatively correlated with NICE scores. While albumin (*r* =  − 0.064, *p* = 0.570), transferrin (*r* =  − 0.135, *p* = 0.230), total cholesterol (*r* =  − 0.141, *p* = 0.210), and lymphocyte counts (*r* =  − 0.160, *p* = 0.154) did not exhibit any significant correlation with NICE scores (Additional file 2: Fig. S1).

### Nutritional factors associated with NTM-LD severity

To identify nutritional factors associated with NTM-LD severity, we performed univariate analysis using low (0) or high (1) NICE scores as the response variable. Given that albumin, transthyretin, retinol-binding protein, and hemoglobin levels were independently associated with disease severity, we performed multiple logistic regression analysis adjusting for age, BMI, and energy intake. The final multiple logistic regression model revealed that transthyretin (odds ratio [OR] 0.872; 95% confidence interval [CI] 0.774 to 0.983, *p* = 0.025) and hemoglobin (OR 0.442; 95% CI 0.259 to 0.756, *p* = 0.003) levels were significantly associated with disease severity (Table 4).

**Table 4:** Univariate and multivariable logistic regression analyses of variables potentially associated with severity of NTM-LD

Variable s	Univariate			Multivariate								
				Model 1			Model 2			Model 3		
	OR	95% CI	*p* Value	OR	95% CI	*p* Value	OR	95% CI	*p* Value	OR	95% CI	*p* Value
Age (years)	1.055	0.998‒1.114	0.057	–	–	–	–	–	–	–	–	–
Body mass index (kg/m^2^)	0.844	0.702‒1.016	0.073	0.848	0.702‒1.024	0.086	–	–	–	–	–	–
Energy intake (kcal/day)	1.000	0.999‒1.001	0.961	1.000	0.999‒1.001	0.851	1.001	0.999‒1.002	0.445	–	–	–
Albumin (g/dL)	0.154	0.027‒0.868	0.034	0.209	0.035‒1.265	0.088	0.286	0.045‒1.825	0.185	0.314	0.047‒2.098	0.232
Transthyretin (mg/dL)	0.843	0.754‒0.943	0.003	0.858	0.765‒0.962	0.008	0.870	0.772‒0.980	0.021	0.872	0.774‒0.983	0.025
Retinol-binding protein (mg/dL)	0.364	0.150‒0.880	0.025	0.368	0.152‒0.893	0.027	0.428	0.168‒1.094	0.076	0.444	0.172‒1.143	0.092
Hemoglobin (g/dL)	0.413	0.244‒0.700	0.001	0.426	0.249‒0.726	0.002	0.441	0.258‒0.753	0.003	0.442	0.259‒0.756	0.003

*NTM-LD* nontuberculous mycobacterial lung disease*, OR* odds ratio, *CI* confidence interval

Determination of NTM-LD severity was based on the median (8.0) NICE score

Model 1: Adjusted for age, Model 2: Adjusted for age and body mass index, Model 3: Adjusted for age, body mass index, and energy intake

## Discussion

Our study had three major findings. First, compared with healthy women, female patients with NTM-LD showed unique characteristics: slender body habitus with low skeletal muscle mass, and smaller intake of energy and macronutrients (protein, fat, and carbohydrate). Second, compared with patients in the mild group, those in the severe group exhibited worsening nutritional biomarkers and low skeletal muscle mass but without an additional decrease in either energy or protein intake. Third, transthyretin and hemoglobin were identified as useful nutritional biomarkers reflecting disease severity.

### Anthropometric and nutritional characteristics of female patients with NTM-LD

Low BMI and less body fat are well-recognized risk factors for NTM-LD [4–6]. Consistent with reports by Kim et al. [5] and Kartalija et al. [6], the present results indicated that female patients with NTM-LD were thinner [5, 6] and had less body fat [6], compared with the general female population [5] or age-matched uninfected women [6]. Additionally, we observed that skeletal muscle mass was lower in the patients than in controls. Since skeletal muscle mass is associated with immune function [21], it is reasonable to assume that reduced skeletal muscle mass may increase susceptibility to NTM infection.

Weight loss or body fat loss is a consequence of negative energy balance; however, information on dietary intake in patients with NTM-LD remains limited. To the best of our knowledge, this is the first report demonstrating that female patients with NTM-LD have lower total energy, protein, fat, and carbohydrate intake than healthy women. Low BMI and aging are both etiological factors for NTM-LD [2–6]. Thus, ameliorating the undernourishment status of women via adequate nutritional intake may be beneficial to prevent NTM-LD, especially in countries and regions with higher proportions of underweight and older people like Japan [22, 23].

### Body composition and NTM-LD severity

In this study, we observed significant decrease in both body weight and skeletal muscle mass in the severe group. The results underscore the need to pay attention to not only weight loss but skeletal muscle loss in the management of NTM-LD. As a clinical implication, skeletal muscle mass has been reported to correlate strongly with respiratory muscle strength metrics, such as maximal inspiratory pressure and maximal expiratory pressure [24]; thus, skeletal muscle loss may be associated with decreased respiratory function [25]. Indeed, lower values of respiratory muscle strength were observed in this study in the severe group. Therefore, strategically planned nutritional care aimed towards the maintenance of skeletal muscle mass may be beneficial for female patients with NTM-LD.

The present study leaves the question “What underpins the differences in skeletal muscle mass, despite the lack of differences in energy and nutrient intake between the mild and severe groups?” One potential factor may be increased energy expenditure due to inefficient breathing. Reports suggest that patients with chronic obstructive pulmonary disease have greater resting energy expenditure owing to increased oxygen cost [26, 27]. Such excess expenditure may partially explain the decrease in skeletal muscle mass. Chronic inflammation related to catabolism is also known to cause a decrease in muscle protein [28, 29]. Although the precise mechanism of skeletal muscle loss in NTM-LD patients should be examined in future studies, maintaining body weight with appropriate muscle mass through sufficient dietary intake might be a beneficial treatment strategy for NTM-LD.

### Nutritional biomarkers and NTM-LD severity

Worsening of hematological and nutritional indices, such as hypoalbuminemia (< 3.5 g/dL) [8–12] and low hemoglobin levels (< 10 g/dL [10] or < 11.3 g/dL [12]) have been reported to be associated with NTM-LD progression and increased mortality [8–12]. Consistent with the above reports, low albumin and hemoglobin levels were also demonstrated in the severe group in this study. The finding that these mid- to long-term nutritional biomarkers were lower in the severe group seems reasonable. However, all our patients had more than 3.5 g/dL of albumin, and the value did not correlate with severity (NICE score) (Additional file 2: Fig. S1). On the other hand, the mean value of transthyretin in the severe group was 18.4 mg/dL, which was lower than the reference value (22–40 mg/dL). For the retinol-binding protein, the mean values were within the reference range (1.9–4.6 mg/dL) but were distributed at lower values, and, like transthyretin, decreased with disease severity (increased NICE score) (Additional file 2: Fig. S1).

Logistic regression analysis was finally performed, revealing that hemoglobin and transthyretin were the nutritional biomarkers that correlated with NTM-LD severity, independent of age, BMI, and energy intake (Table 4). Thus, hemoglobin may be a useful indicator of long-term malnutrition and transthyretin (half-life, 2 days) as a beneficial indicator of short-term malnutrition, at least in the outpatients enrolled in the study. Transthyretin is also an indicator of dynamic protein synthesis capacity [30–32]. To clarify the cause of muscle protein loss in NTM-LD patients, further measurement of urinary 3-methylhistidine excretion, which reflects muscle protein breakdown, in addition to muscle protein synthesis indices, may be required.

## Limitations

This study has some limitations. First, because of the cross-sectional design, it remains unclear whether slender body habitus observed in female patients with NTM-LD is a cause or a consequence of the disease. In the future, it is necessary to obtain consecutive pre- and post-infection weight data to clarify whether low body weight itself is a risk factor for the development of the disease or whether weight loss occurs as a result of the disease. Second, homogeneity constituted a strength of this study, although there might have been selection bias, as all participants were Japanese, similar-aged women and were recruited from the same hospital and school. Third, we failed to clarify the kinetics of muscle protein synthesis and breakdown due to the lack of urinary 3-methylhistidine data.

## Conclusions

Eating less and slender body habitus with low skeletal muscle mass were identified as nutritional characteristics of female patients with NTM-LD. In patients with severe symptoms, further reductions in body weight and skeletal muscle mass were observed, without additional reduction in nutritional intake. This is the first study to comprehensively report the association between NTM-LD severity and nutritional status, including body composition, nutrient intake, and biomarkers. The results suggest that initiating nutritional therapy from the mild stage of the disease to prevent undernutrition is warranted.

## Supplementary Information

**Additional file 1.** Anthropometric and nutritional data of female patients with NTM-LD and age- and sex-matched healthy controls.

**Additional file 2.** Correlations between NICE score and biochemical markers.

## Data Availability

The datasets generated and/or analyzed during the current study are not publicly available due to their containing information that could compromise the privacy of research participants but are available from the corresponding author upon reasonable request.

## Abbreviations

NTM-LD: Nontuberculous mycobacterial lung disease
BMI: Body mass index
ATS: American Thoracic Society
IDSA: Infectious Diseases Society of America
NICE: Nodule, infiltration or consolidation, cavity, ectasis
SNAQ-J: Japanese version of Simplified Nutritional Appetite Questionnaire
IPAQ: International standardized Physical Activity Questionnaire
