# Supplementary material for: Nutritional status in female patients with nontuberculous mycobacterial lung disease and its association with disease severity
Source: BMC Pulm Med. 2022 Aug 15;22:315. doi: 10.1186/s12890-022-02109-5 (PMC9380388; doi:10.1186/s12890-022-02109-5)
Supplement: Supplementary file 1 — Additional file 1. Anthropometric and nutritional data of female patients with NTM-LD and age- and sex-matched healthy controls. [file 12890_2022_2109_MOESM1_ESM.docx]

**Table S1** Anthropometric and nutritional data of female patients with NTM-LD

and age- and sex-matched healthy controls

| Variables | NTM-LD (*n* = 38) | Control (*n* = 38) | *p* value ^a^ |
| --- | --- | --- | --- |
| Age (years) | 71.6 ± 3.9 | 71.5 ± 3.5 | 0.878 |
| Anthropometric data |  |  |  |
| Height (cm) | 154.2 ± 6.2 | 152.8 ± 5.6 | 0.314 |
| Body weight (kg) | 46.2 ± 6.3 | 53.6 ± 7.8 | < 0.001 |
| Body mass index (kg/m^2^) | 19.4 ± 2.6 | 23.0 ± 2.8 | < 0.001 |
| Fat mass (kg) | 12.3 ± 4.8 | 16.7 ± 5.4 | < 0.001 |
| Percentage of body fat (%) | 25.8 ± 7.7 | 30.6 ± 6.4 | 0.004 |
| Skeletal muscle mass (kg) | 17.7 ± 2.0 | 19.7 ± 2.4 | < 0.001 |
| Nutritional data |  |  |  |
| Energy (kcal/day) | 1,717 ± 382 | 1,914 ± 327 | 0.018 |
| Protein (g/day) | 65.1 ± 17.3 | 79.1 ± 16.9 | 0.001 |
| Fat (g/day) | 52.1 ± 17.2 | 62.2 ± 15.5 | 0.009 |
| Carbohydrate (g/day) | 241.4 ± 64.1 | 252.1 ± 61.5 | 0.459 |
| Mean ± SD, ^a^ Unpaired t-test  *NTM-LD* nontuberculous mycobacterial lung disease | | |  |
