# Supplementary material for: Nutritional status in female patients with nontuberculous mycobacterial lung disease and its association with disease severity
Source: BMC Pulm Med. 2022 Aug 15;22:315. doi: 10.1186/s12890-022-02109-5 (PMC9380388; doi:10.1186/s12890-022-02109-5)
Supplement: Supplementary file 2 — Additional file 2. Correlations between NICE score and biochemical markers. [file 12890_2022_2109_MOESM2_ESM.docx]

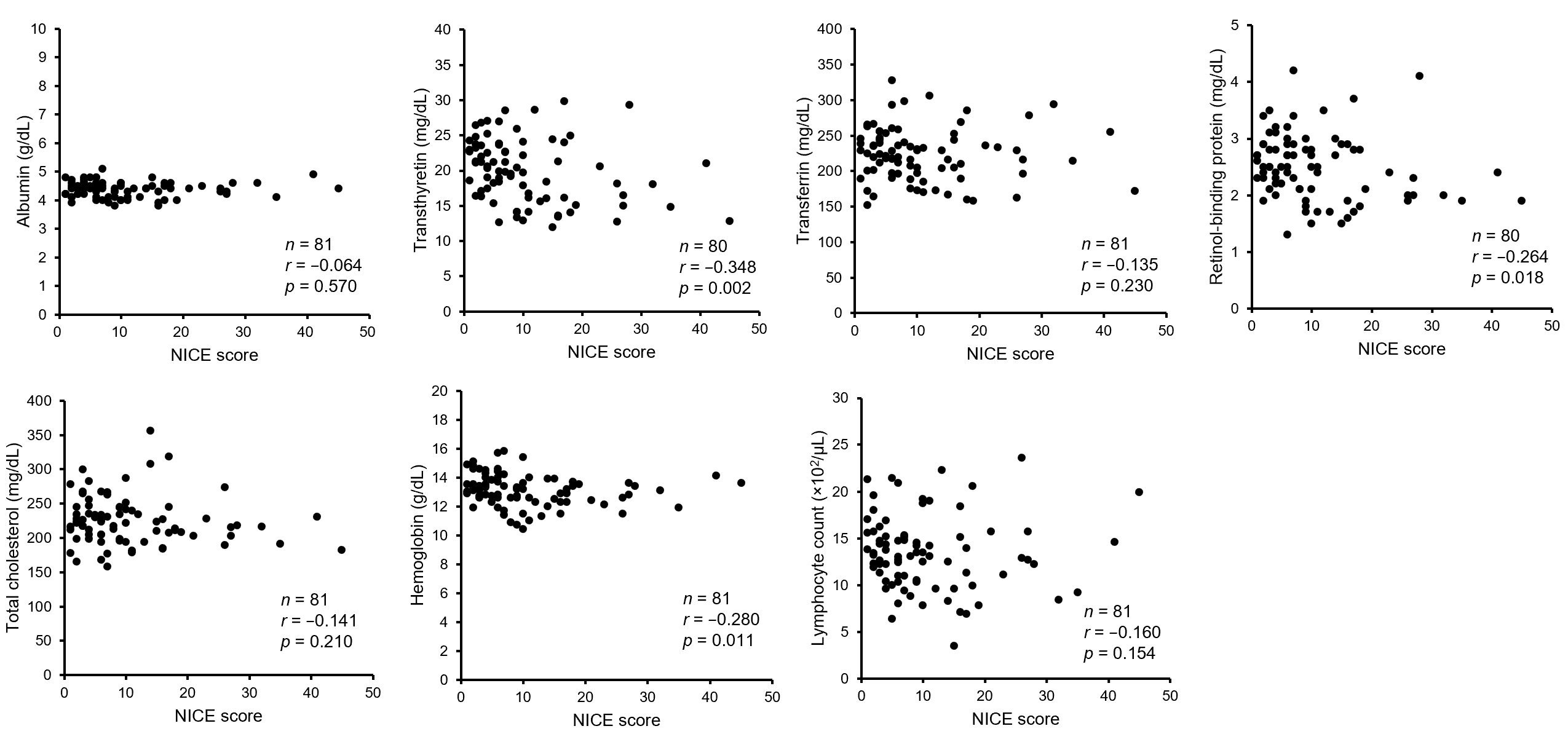


**Figure S1** Correlations between NICE score and biochemical markers

*Spearman’*s correlation

Transthyretin and retinol-binding protein for one patient could not be obtained.

*NICE score* nodule, infiltration or consolidation, cavity, ectasis score
